# Supplementary material for: Extrinsic hydrophobicity-controlled silver nanoparticles as efficient and stable catalysts for CO2 electrolysis
Source: Nat Commun. 2024 Apr 18;15:3356. doi: 10.1038/s41467-024-47490-3 (PMC11026478; doi:10.1038/s41467-024-47490-3)
Supplement: Supplementary file 1 — Supplementary Information [file 41467_2024_47490_MOESM1_ESM.pdf]

Supporting Information for

Extrinsic hydrophobicity-controlled silver nanoparticles as efficient and stable catalysts for CO_2 electrolysis

Young-Jin Ko,[1,†,] Chulwan Lim,[1,2,†] Junyoung Jin,[3,4,†] Min Gyu Kim,[5] Ji Yeong Lee,[6] Tae-Yeon Seong,[4] Kwan-Young Lee,[2] Byoung Koun Min,[1] Jae-Young Choi,[7,8] Taegeun Noh,[9] Gyu Weon Hwang,[3,] Woong Hee Lee,[1,]* Hyung-Suk Oh[1,7,8,]**

[1] Clean Energy Research Center, Korea Institute of Science and Technology (KIST), Hwarang-ro 14-gil 5, Seongbuk-gu, Seoul 02792, Republic of Korea

[2] Department of Chemical and Biological Engineering, Korea University, 145, Anam-ro, Seongbuk-gu, Seoul 02841, Republic of Korea

[3] Center for Neuromorphic Engineering, Korea Institute of Science and Technology (KIST), Hwarang-ro 14-gil 5, Seongbuk-gu, Seoul 02792, Republic of Korea

[4] Department of Materials Science and Engineering, Korea University, 145, Anam-ro, Seongbuk-gu, Seoul 02841, Republic of Korea

[5] Beamline Research Division, Pohang Accelerator Laboratory (PAL), Pohang 37673, Republic of Korea

[6] Advanced Analysis Center, Korea Institute of Science and Technology (KIST), Hwarang-ro 14-gil 5, Seongbuk-gu, Seoul 02792, Republic of Korea

[7] School of Advanced Materials Science & Engineering, Sungkyunkwan University (SKKU), Suwon, 16419, Republic of Korea

[8] KIST-SKKU Carbon-Neutral Research Center, Sungkyunkwan University (SKKU), Suwon 16419, Republic of Korea

[9] Platform Technology Research Center, LG Chem Ltd., 30, Magokjungang 10-ro, Gangseo-gu, Seoul 07796, Republic of Korea

† All authors contributed equally to this work.

***Corresponding Authors**

E-mail address: gwhwang@kist.re.kr (G. W. Hwang), abcabac@kist.re.kr (W. H. Lee), hyung-suk.oh@kist.re.kr (H.-S. Oh)

Tel.: +82 (0)2 958 5292

Table of Contents

Contents	Page

○ **Supplementary Materials** ………………………………………………………

□ Supplementary Figures 1–29 S3-S25, S27, S30, S33-S36

□ Supplementary Table 1–3 S26, S28, S31

□ Supplementary Note 1 S32

○ **Supplementary Movies** ……………………………………………………… Attached files

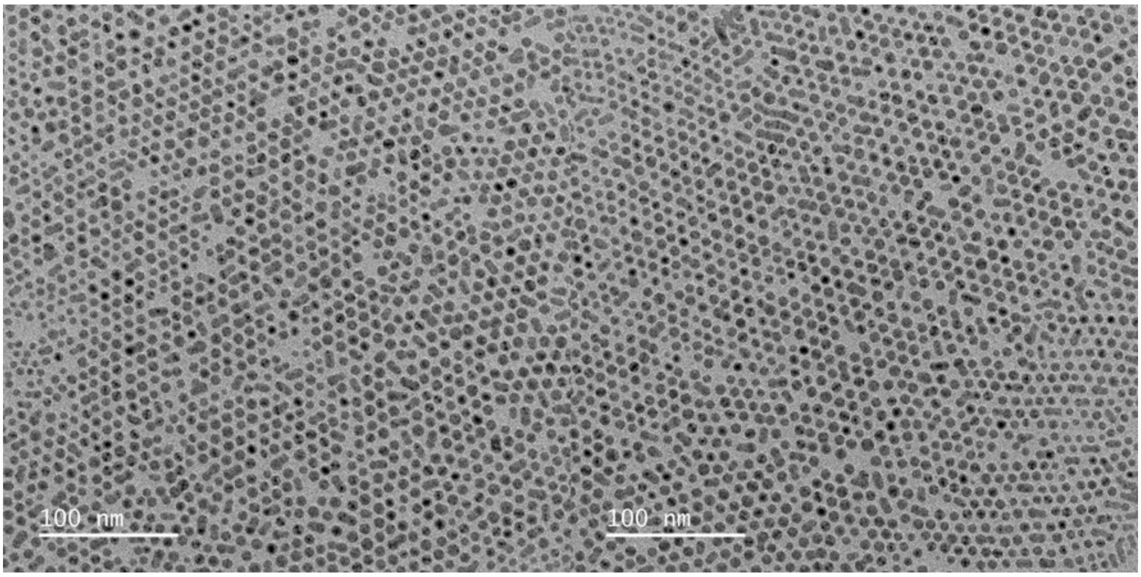

Supplementary Figure 1. low-magnitude TEM images of Ag-NP catalyst with 2 h of tetramethylammonium hydroxide treatment

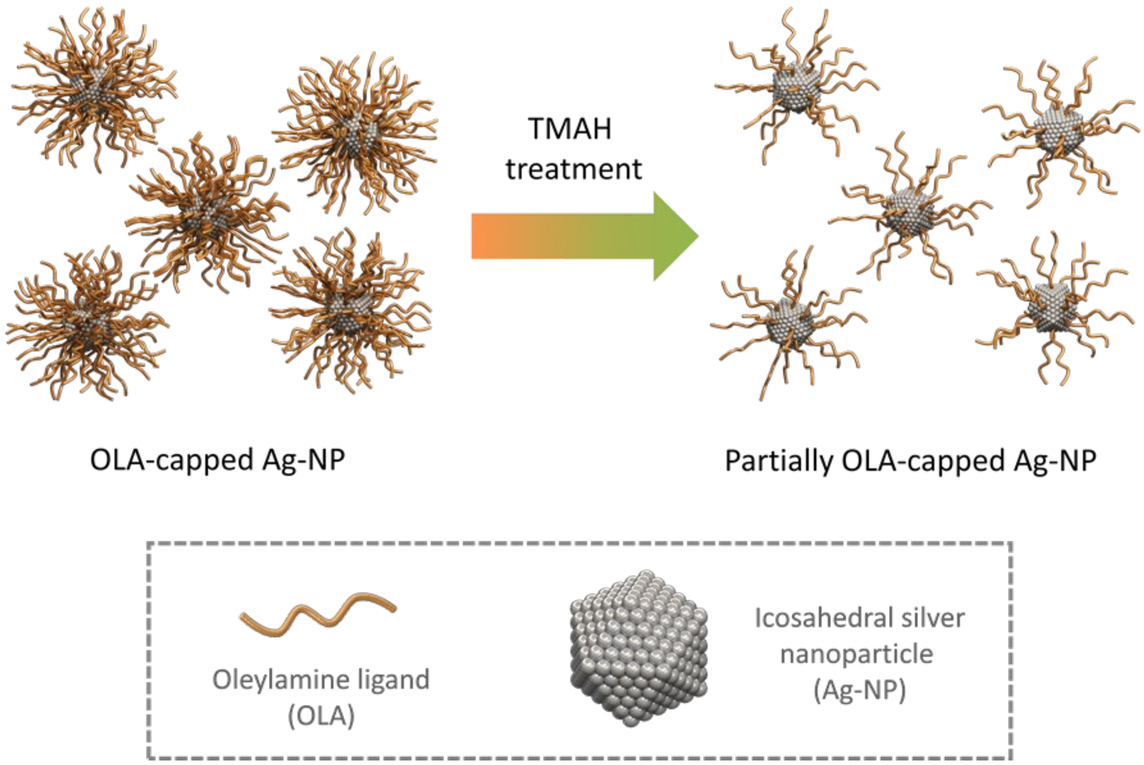

Supplementary Figure 2. Schematic diagram of the synthesis process of tetramethylammonium hydroxide (TMAH) treated Ag-NP catalysts.

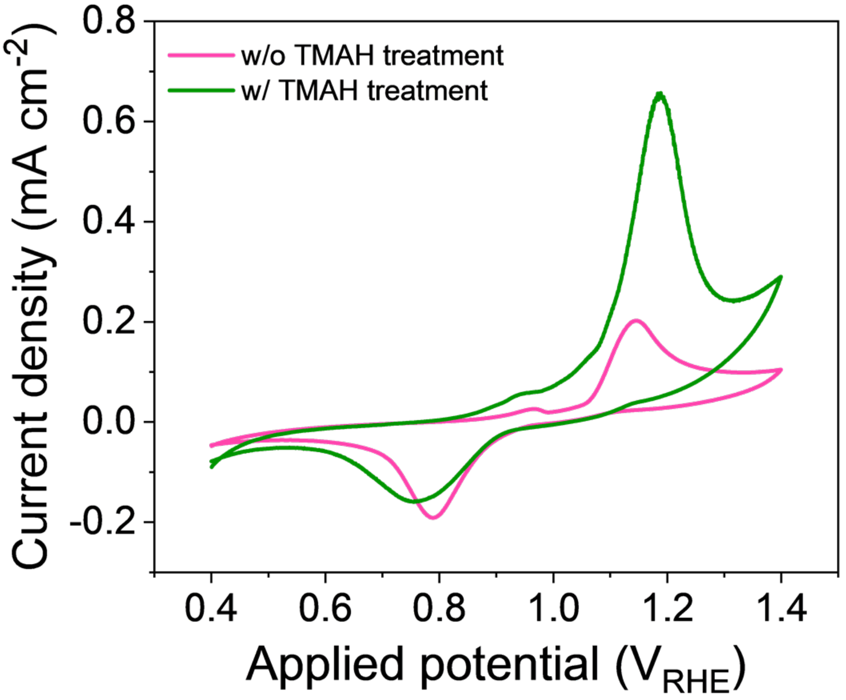

Supplementary Figure 3. Cyclic voltammograms of Ag-NP catalysts with and without 2 h of tetramethylammonium hydroxide (TMAH) treatment in 0.1 M $KHCO_3$ solution obtained at a scan rate of 50 mV s^{-1}.

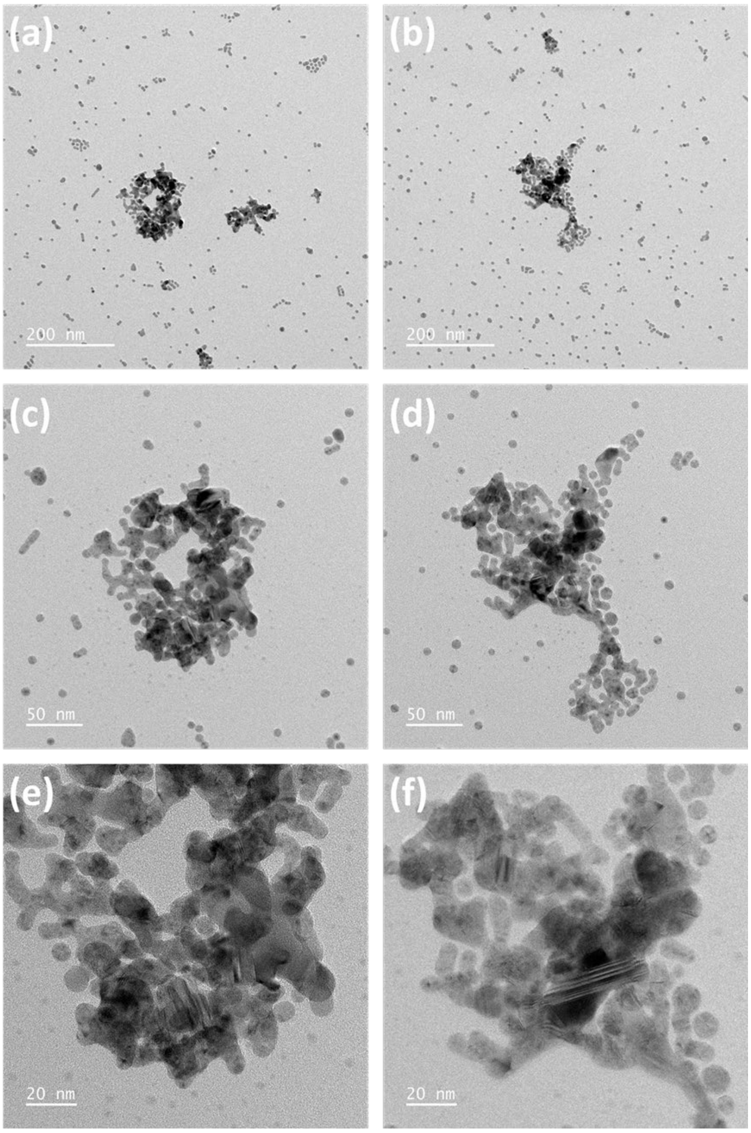

Supplementary Figure 4. (a-d) low-magnitude TEM images and (e-f) HR-TEM images of Ag-NP catalyst with 5 h of tetramethylammonium hydroxide treatment.

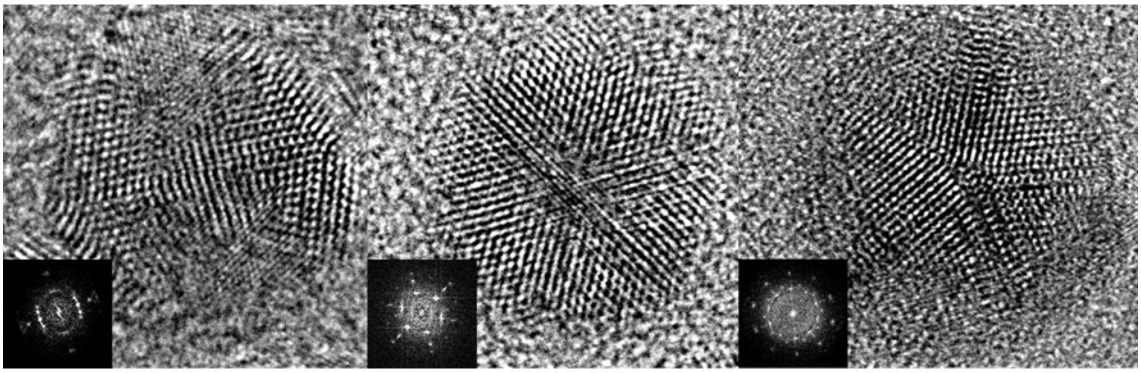

Supplementary Figure 5. HR-TEM images of Ag-NP catalyst with 2 h of tetramethylammonium hydroxide treatment for 2, 3, and 5-fold verification; scale bar = 3 nm.

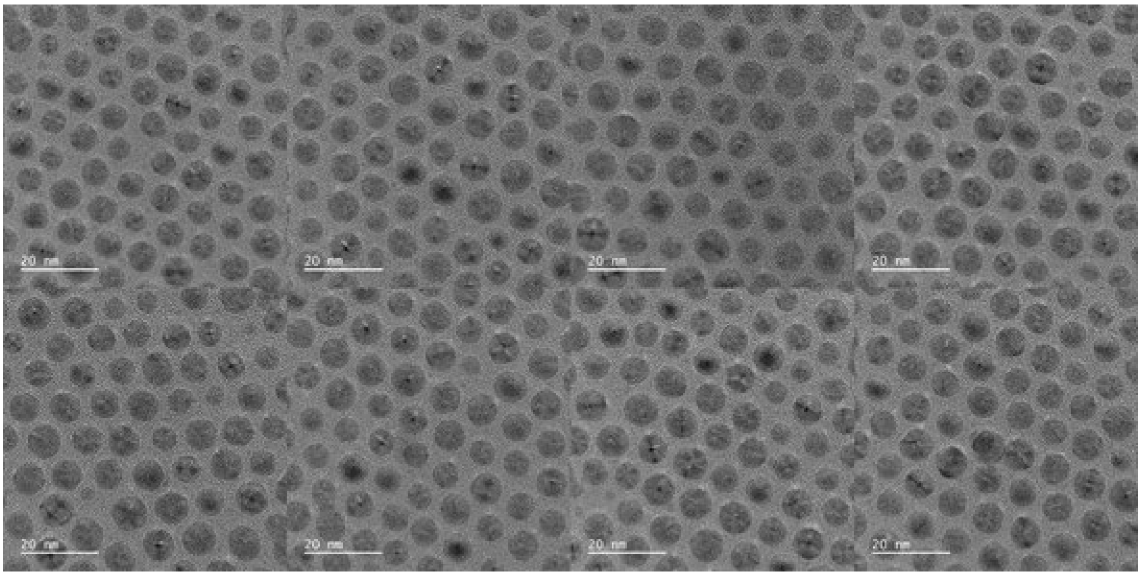

Supplementary Figure 6. HR-TEM images of Ag-NP catalyst with 2 h of tetramethylammonium hydroxide treatment for calculation of particle size distribution and average particle size.

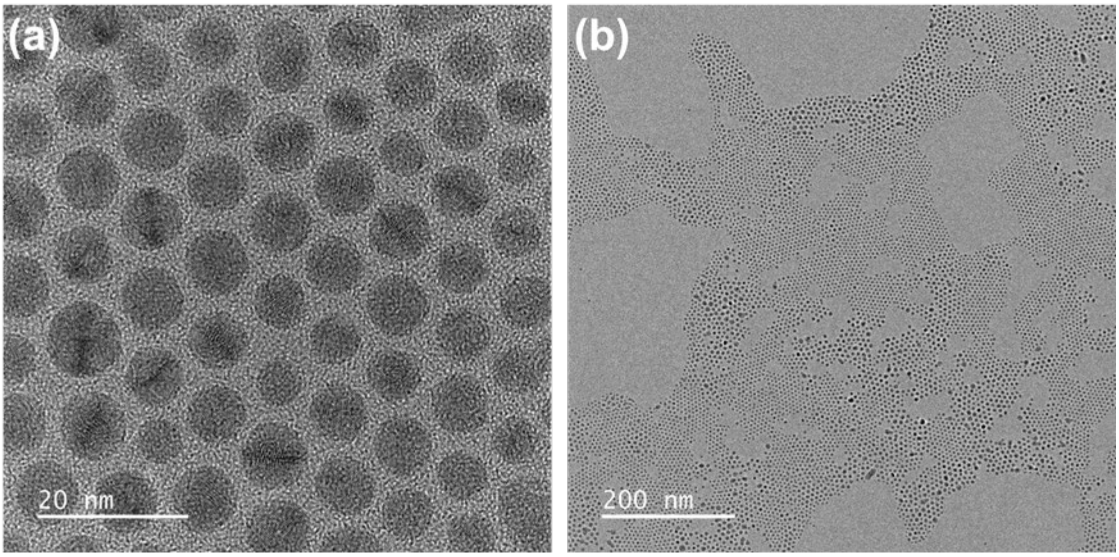

Supplementary Figure 7. (a) HR-TEM image and (b) low-magnitude TEM image of pristine Ag-NP catalyst.

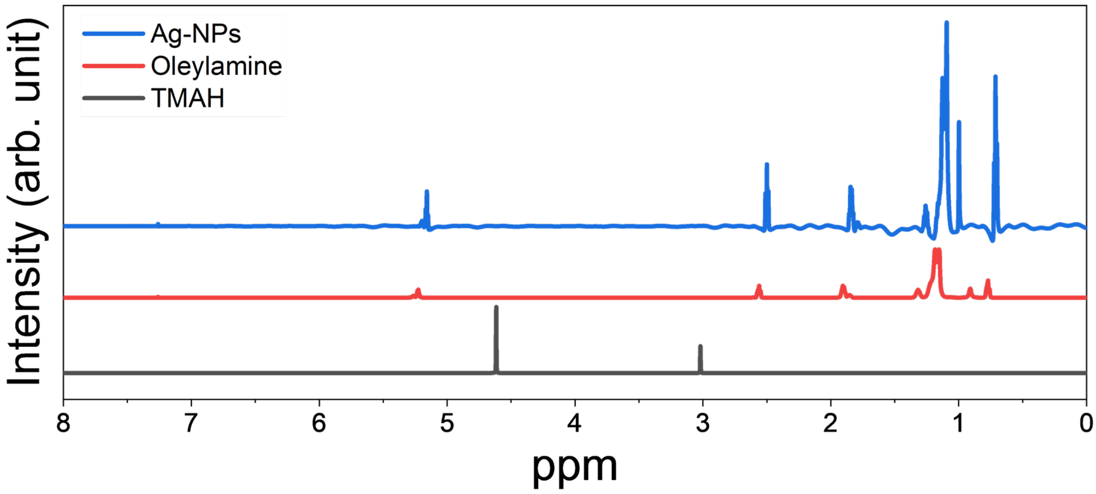

Supplementary Figure 8. ^{1}H NMR spectra of Ag-NP, Oleylamine and tetramethylammonium hydroxide (TMAH).

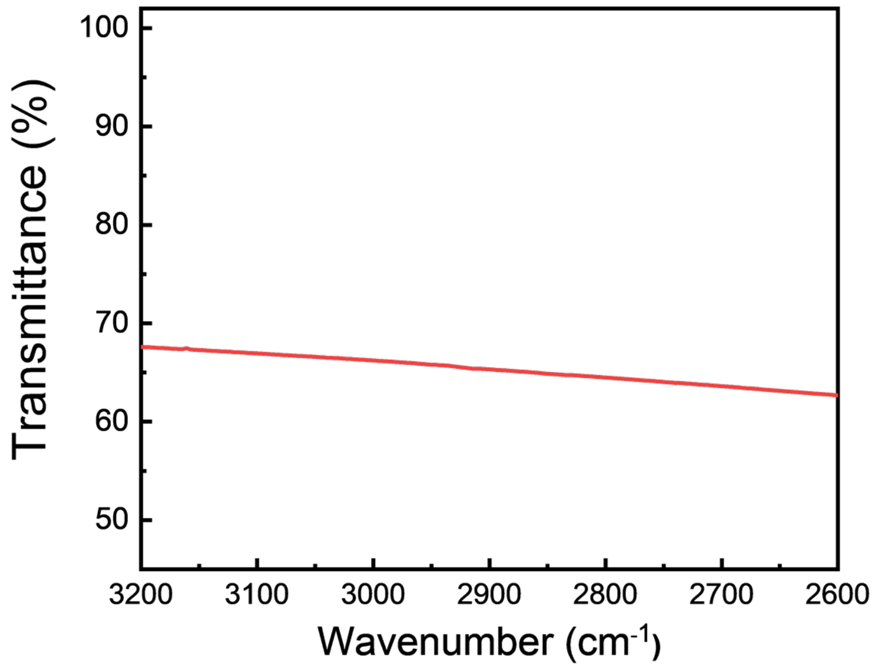

Supplementary Figure 9. FT-IR spectra (2600-3200 cm^{-1}) of the GDL.

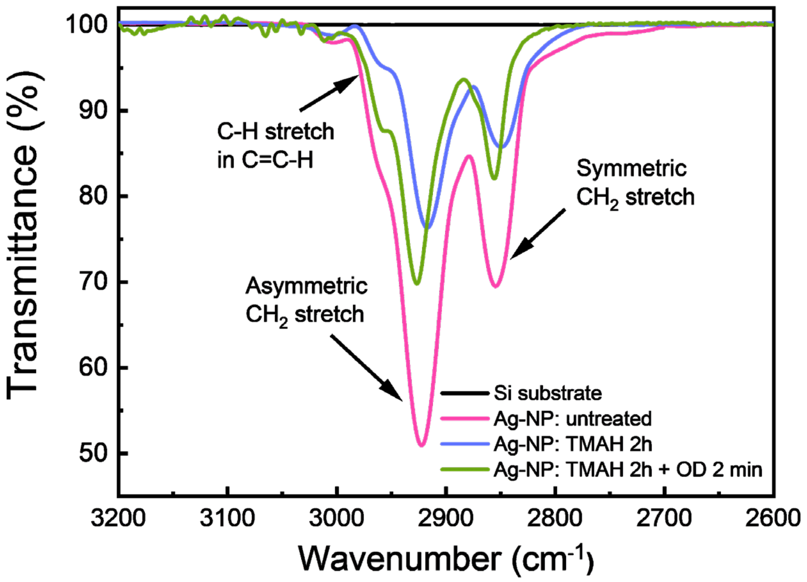

Supplementary Figure 10. FT-IR spectra (2600-3200 cm^{-1}) of as-prepared and tetramethylammonium hydroxide (TMAH) treated Ag-NP electrodes. The lipid ligand wasn't detached even in the pre-oxidation reaction for 2 min, which is the same as actual CO_2RR test.

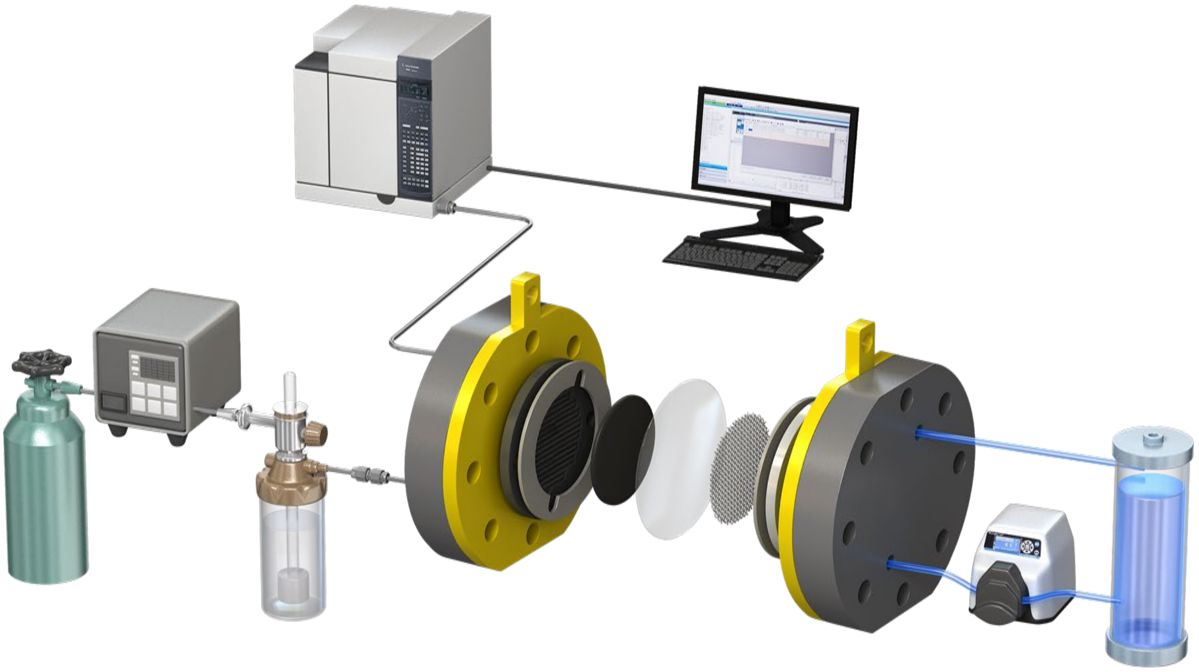

Supplementary Figure 11. Schematic of electrochemical CO_2 reduction experimental setup.

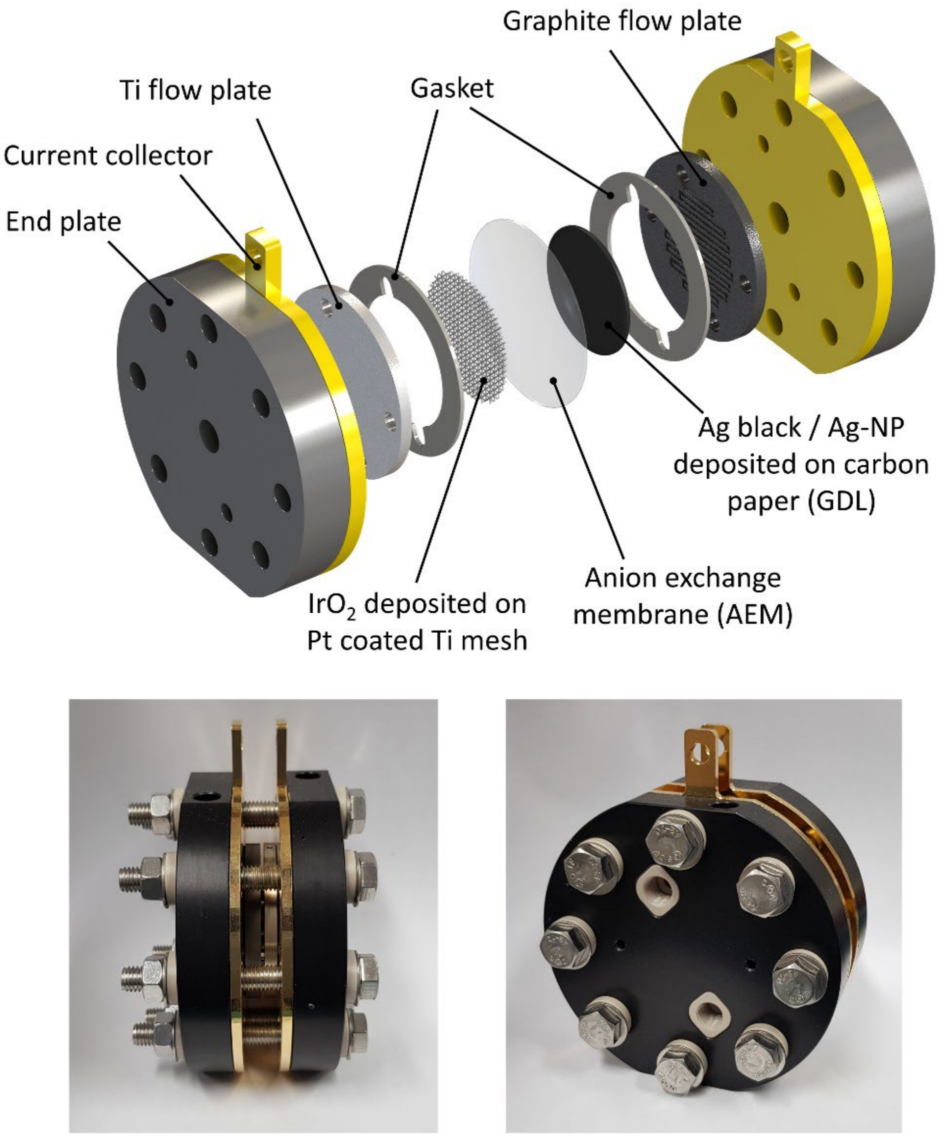

Supplementary Figure 12. The components and assembly of the zero-gap CO_2 electrolyzer system using a gas diffusion layer (GDL).

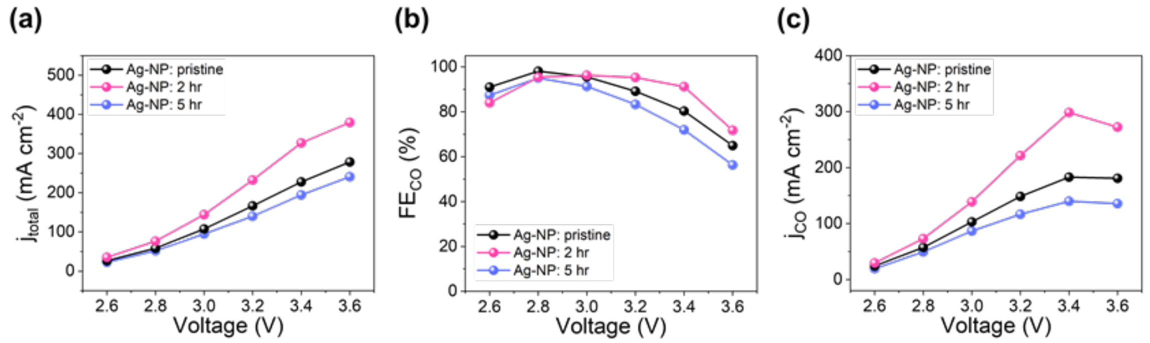

Supplementary Figure 13. CO_2RR performance for Ag-NP catalysts with various tetramethylammonium hydroxide (TMAH) treatment time in 0.1 M $KHCO_3$ electrolyte. (a) Total current density, (b) CO faradaic efficiency, and (c) CO partial current density.

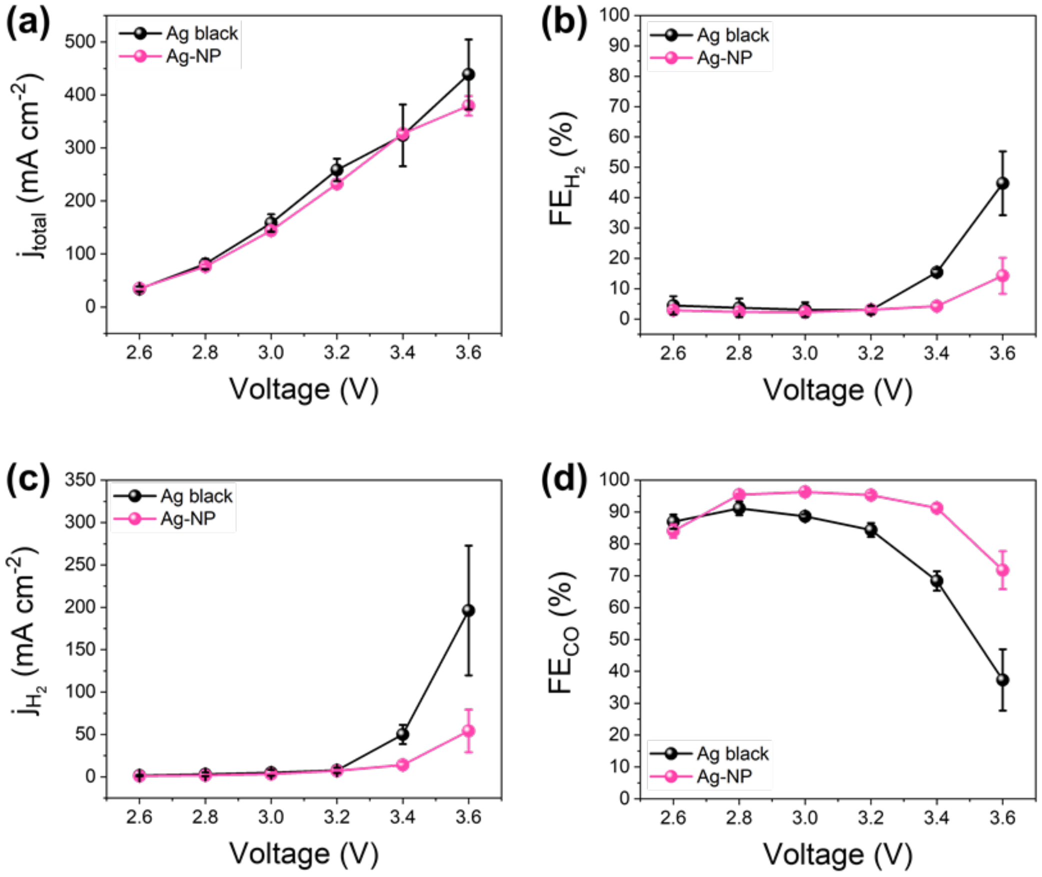

Supplementary Figure 14. CO_2RR performance and HER measured for Ag black and Ag-NP catalysts in 0.1 M $KHCO_3$ electrolyte. Each experiment was measured three times to obtain the average value and error. (a) Total current density, (b) H_2 faradaic efficiency, (c) H_2 partial current density, and (d) CO faradaic efficiency.

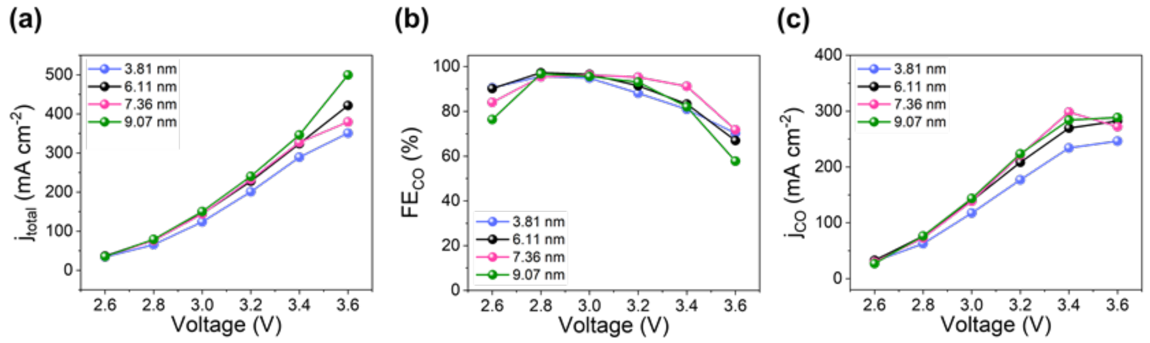

Supplementary Figure 15. CO_2RR performance measured for Ag-NP catalysts with various particle size in 0.1 M $KHCO_3$ electrolyte. (a) Total current density, (b) CO faradaic efficiency, and (c) CO partial current density.

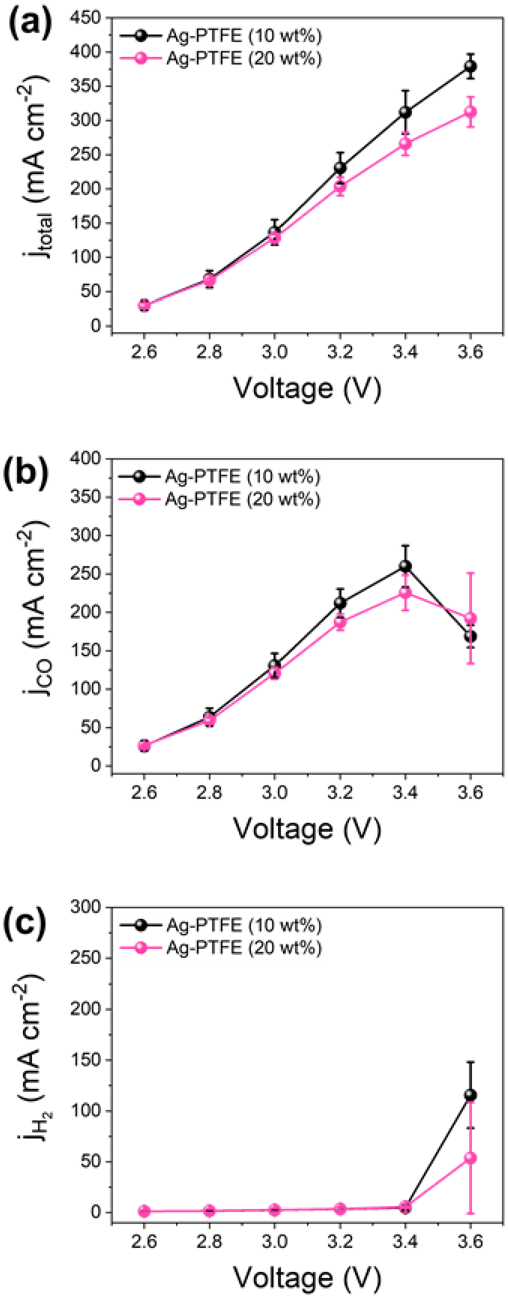

Supplementary Figure 16. CO_2RR performance and HER measured for Ag-Polytetrafluoroethylene (PTFE) catalysts in 0.1 M $KHCO_3$ electrolyte. Each experiment was measured three times to obtain the average value and error. (a) Total current density, (b) CO partial current density, and (c) H_2 partial current density.

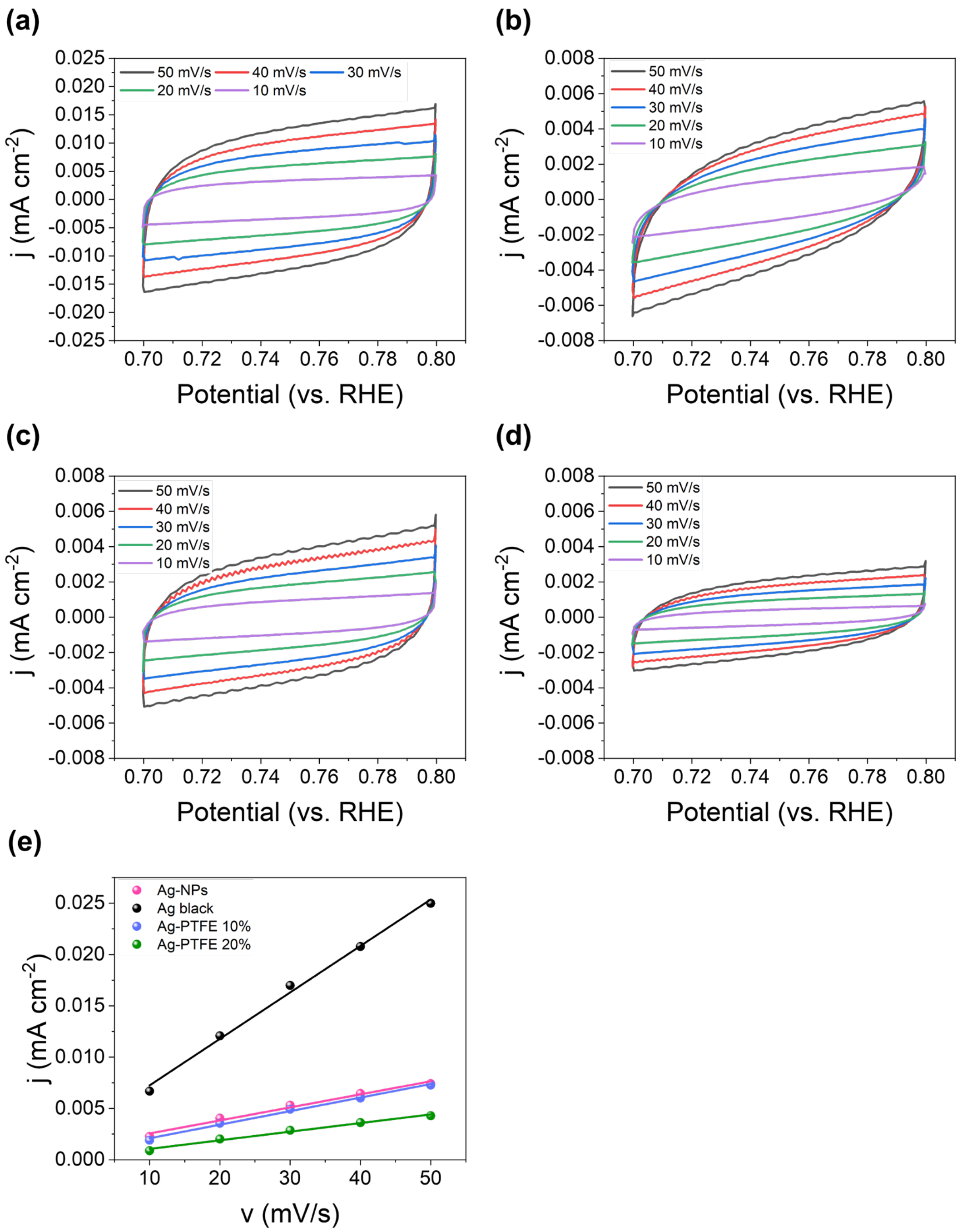

Supplementary Figure 17. CV measured for (a) Ag black, (b) Ag-NP, (c) Ag-Polytetrafluoroethylene (PTFE) 10%, and (d) Ag-PTFE 20% catalysts in non-Faradaic potential range at various scan rate. (e) Linear fitting of the capacitive currents versus CV scan rates.

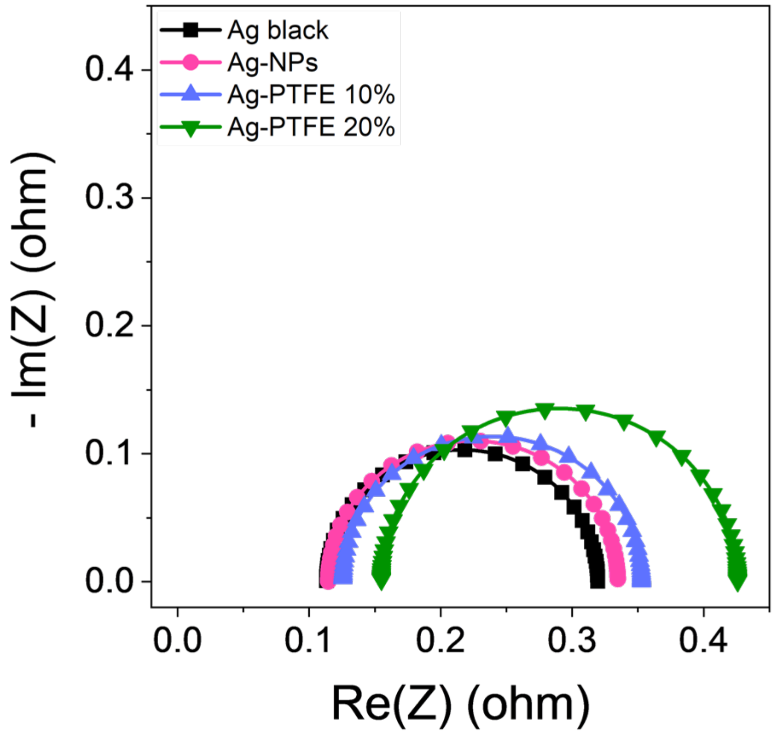

Supplementary Figure 18. Electrochemical impedance spectra for Ag black, Ag-NP, Ag-Polytetrafluoroethylene (PTFE) 10%, and Ag-PTFE 20% catalysts at a cell voltage of -3 V from 10^5 Hz down to 1 Hz with 10 mV AC amplitude.

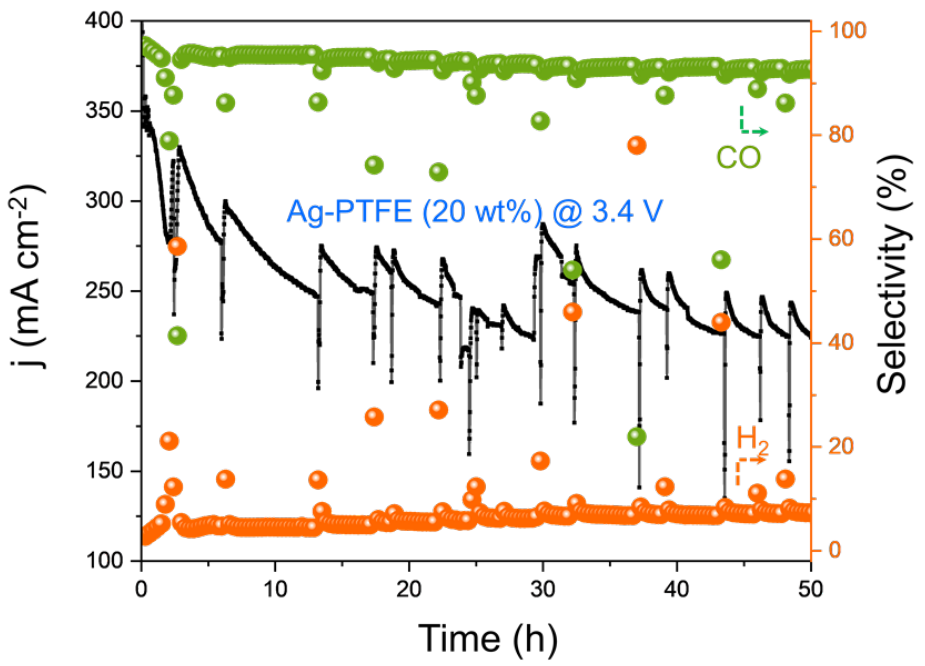

Supplementary Figure 19. Durability test of the Ag-Polytetrafluoroethylene (PTFE) (20 wt%) catalyst in the zero-gap CO_2 electrolyzer at 3.4 V for 50 h. Faradaic efficiency and selectivity of CO and H_2 measured during the durability tests. Electrodes of the CO_2 electrolyzer were prepared with 0.3 mg cm^{-2} of Ag catalysts on 10-cm^2 GDL in the cathode side.

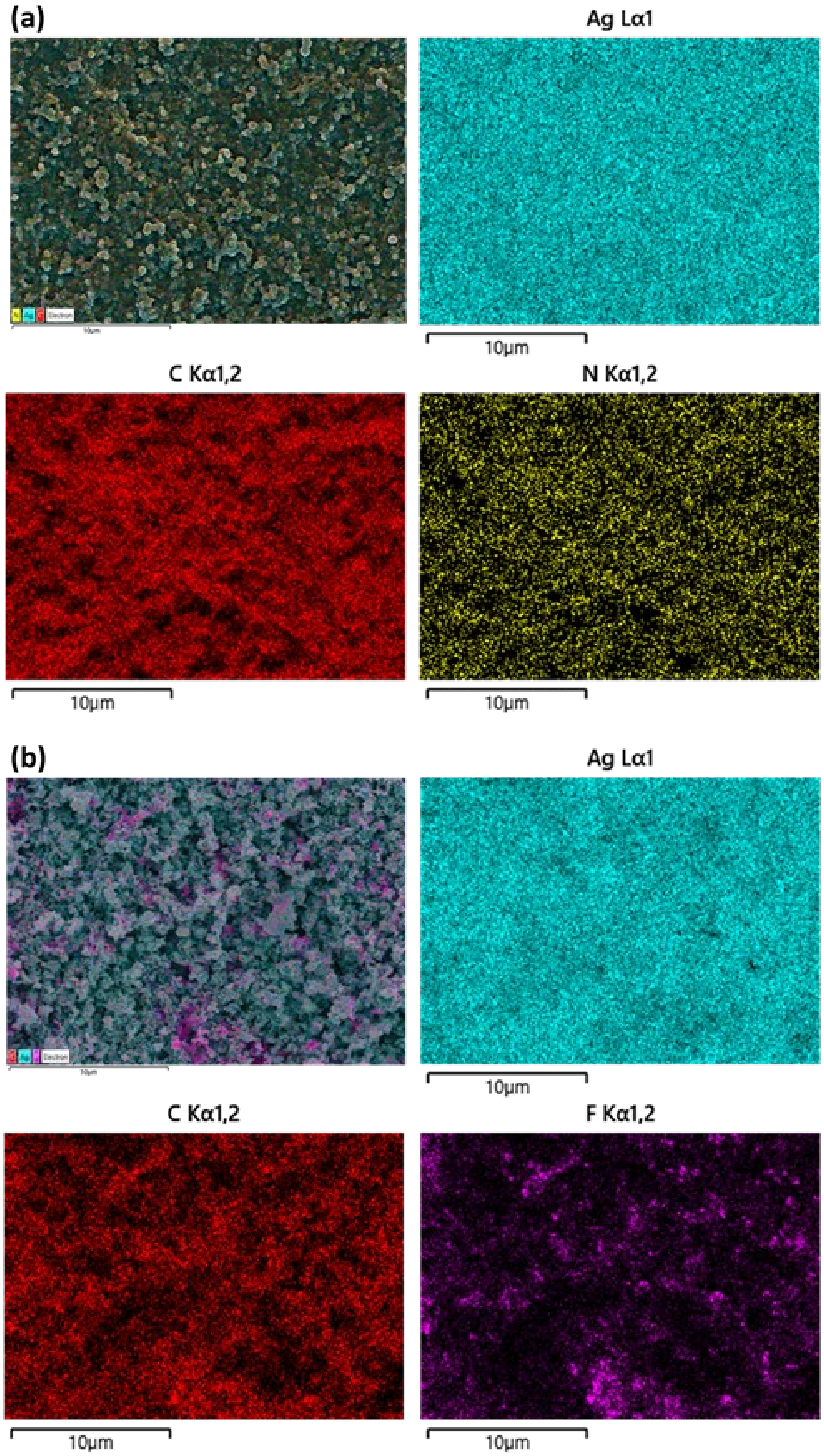

Supplementary Figure 20. EDS mapping images of (a) Ag-NP and (b) Ag-Polytetrafluoroethylene (PTFE) electrodes.

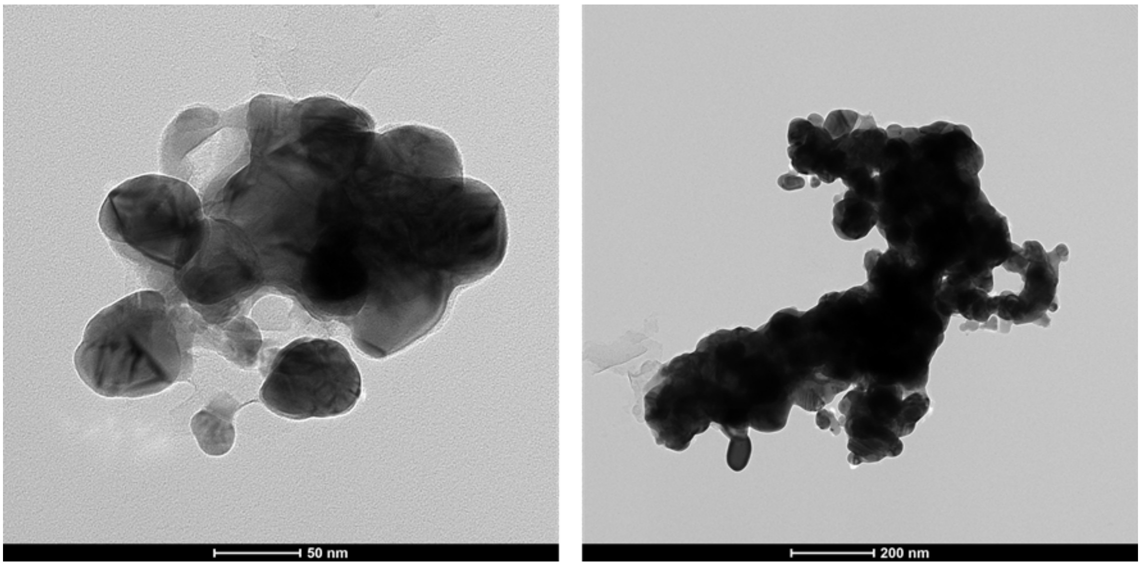

Supplementary Figure 21. HR-TEM images of Ag black catalyst after durability test at 3.4 V for 15 h in zero-gap electrolyzer.

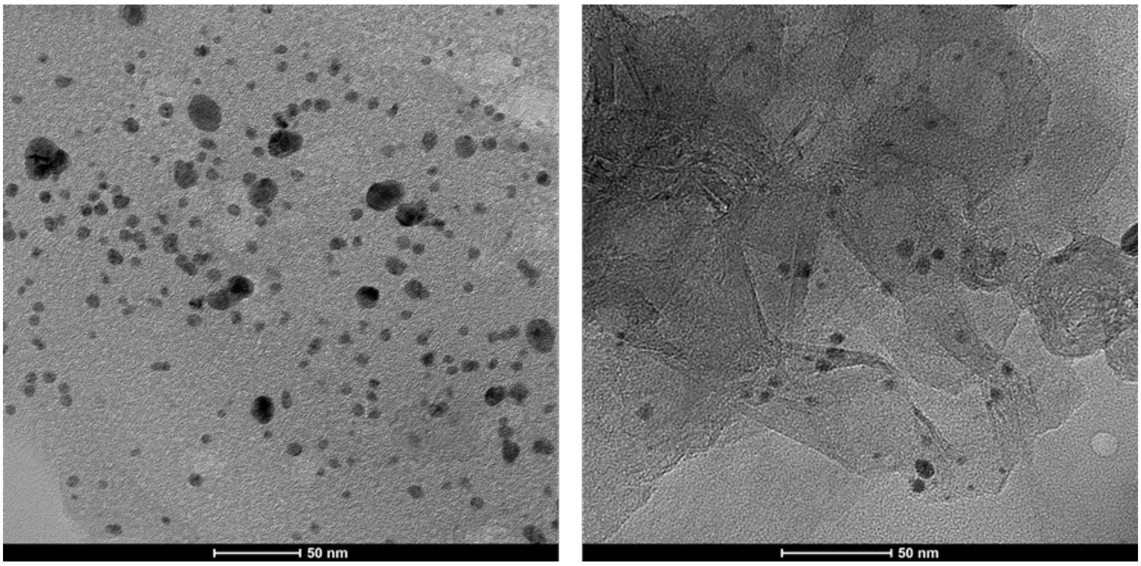

Supplementary Figure 22. HR-TEM images of Ag-NP catalyst after durability test at 3.4 V for 50 h in zero-gap electrolyzer.

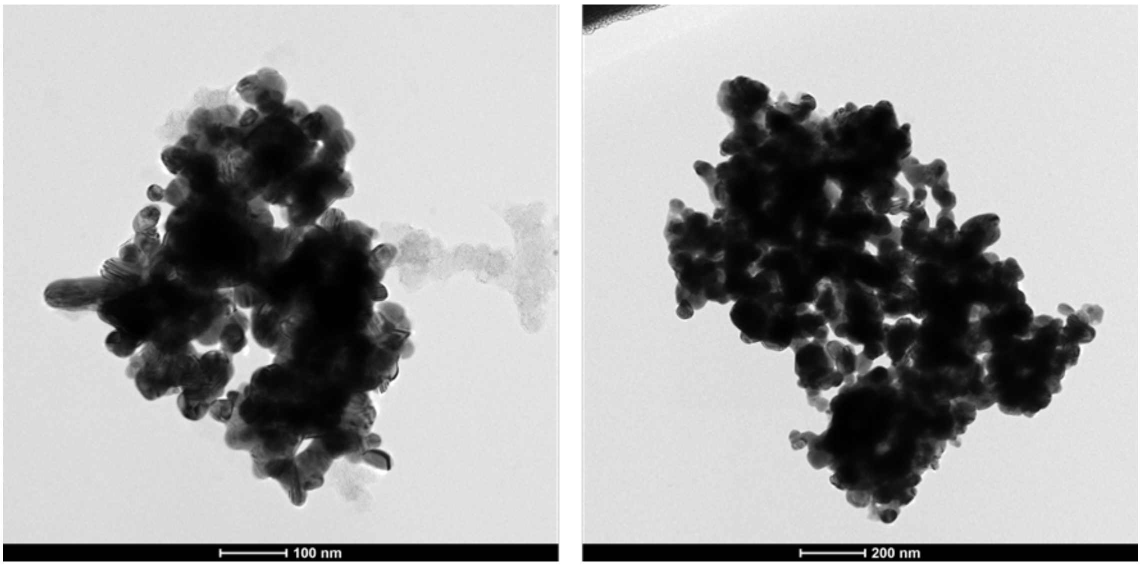

Supplementary Figure 23. HR-TEM images of Ag-Polytetrafluoroethylene (PTFE) catalyst after durability test at 3.4 V for 50 h in zero-gap electrolyzer.

Supplementary Table 1. Cathode potential in a zero-gap electrolyzer experiment for Ag black catalyst.

Total Cell voltage (V)	Current density (mA cm^{-2})	Anode Voltage (V)	Cathode Voltage (V)
2.6	51.1277	1.07128	1.52872
2.8	97.2284	1.1166	1.6834
3.0	174.987	1.16315	1.83685
3.2	279.084	1.20479	1.99521
3.4	355.273	1.25557	2.14443
3.6	417.298	1.30137	2.29863

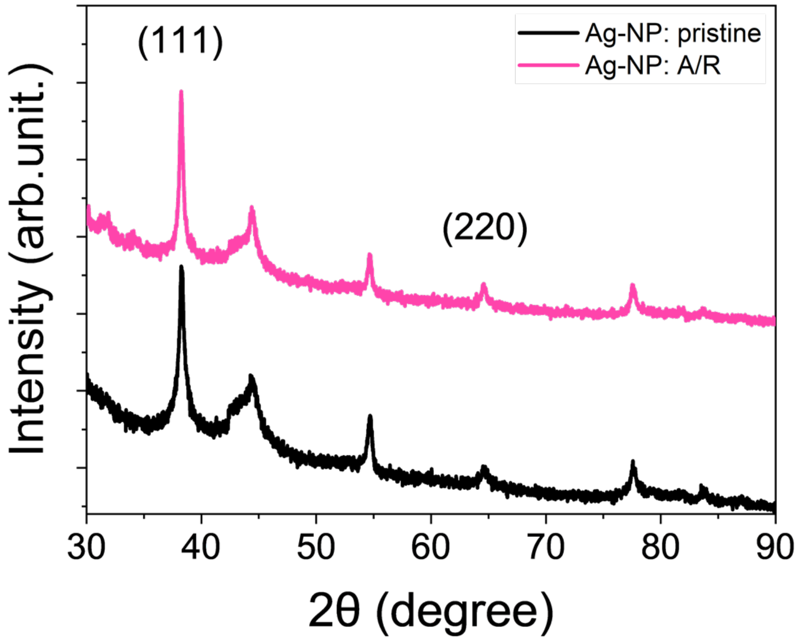

Supplementary Figure 24. Wide-angle XRD patterns before and after CO_2RR for Ag-NP catalysts.

Supplementary Table 2. Summary of experimental result for AEM zero-gap-type CO_2 electrolyzer.

Ag catalyst	Cell potential (V)	Total current density (mA cm^{-2})	H_2 F.E. (%)	CO F.E. (%)	H_2 Partial Current density (mA cm^{-2})	CO Partial Current density (mA cm^{-2})	H_2 selectivity (%)	CO selectivity (%)
Ag black	2.6	33.98	4.49	86.95	1.53	29.54	4.91	95.09
	2.8	81.17	3.71	91.15	3.01	73.99	3.91	96.09
	3.0	158.37	3.08	88.63	4.88	140.37	3.36	96.64
	3.2	258.56	2.98	84.36	7.72	218.12	3.42	96.58
	3.4	323.64	15.45	68.36	50.01	221.22	18.44	81.56
	3.6	438.73	44.74	37.3	196.28	163.63	54.54	45.46
Ag-NP	2.6	35.12	2.92	84.03	1.02	29.51	3.35	96.65
	2.8	76.39	2.38	95.41	1.82	72.89	2.43	97.57
	3.0	144.09	2.28	96.29	3.28	138.75	2.31	97.69
	3.2	232.35	3.08	95.30	7.16	221.43	3.13	96.87
	3.4	327.09	4.28	91.22	14.01	298.39	4.48	95.52
	3.6	379.51	14.27	71.74	54.17	272.28	16.59	83.41
Ag-PTFE (10 wt%)	2.6	30.18	3.95	86.56	1.19	26.12	4.36	95.64
	2.8	68.18	2.41	93.32	1.64	63.62	2.52	97.48
	3.0	136.70	1.83	95.67	2.50	130.78	1.87	98.13
	3.2	230.66	1.54	91.93	3.54	212.04	1.64	98.36

	3.4	312.00	1.54	83.3	4.79	259.91	1.81	98.19
	3.6	379.22	30.49	44.5	115.62	168.76	40.66	59.34
Ag-PTFE (20 wt%)	2.6	30.26	4.03	88.51	1.22	26.78	4.36	95.64
	2.8	66.23	2.56	89.48	1.7	59.26	2.78	97.22
	3.0	128.68	1.95	93.83	2.51	120.74	2.04	97.96
	3.2	203.69	1.73	91.95	3.53	187.30	1.85	98.15
	3.4	266.01	2.11	84.84	5.61	225.70	2.42	97.58
	3.6	312.67	17.17	61.48	53.70	192.22	21.84	78.16

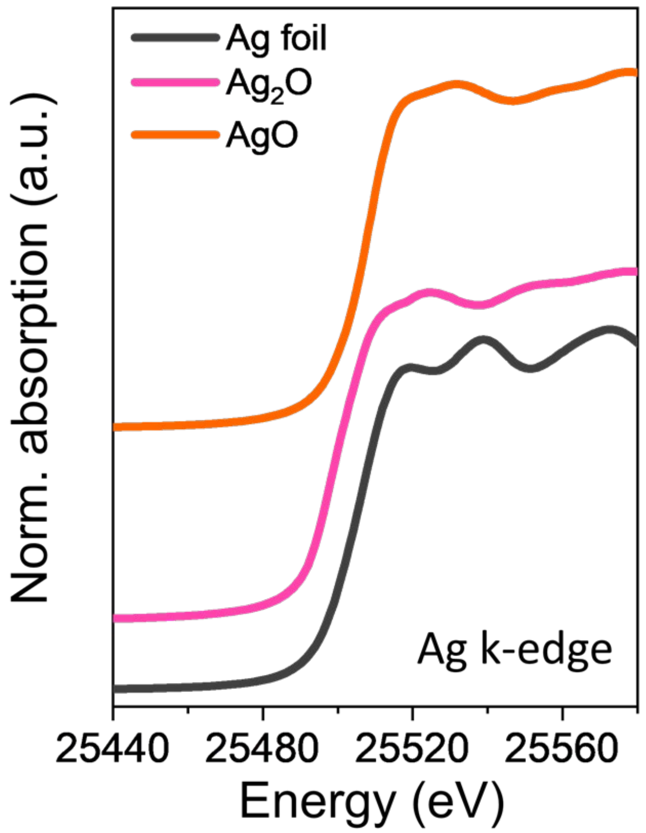

Supplementary Figure 25. XANES spectra at the Ag k-edge for the Ag foil, Ag(I) oxide, and Ag(II) oxide references.

Supplementary Table 3. XANES fitting results of Ag black and Ag-NP catalysts.

	Ag black			Ag-NP		
	Ag^0	Ag^{1+}	Ag^{2+}	Ag^0	Ag^{1+}	Ag^{2+}
ex-situ	0.886	0.112	0.020	0.823	0.159	0.018
OD	0.383	0.488	0.129	0.513	0.392	0.950
2.6 V	0.672	0.265	0.063	0.655	0.304	0.041
3.4 V	0.772	0.176	0.052	0.700	0.262	0.038

Supplementary Note 1. XANES analysis: Linear combination fitting (LCF)

The general approach to XANES analysis is to treat measured XANES data as a linear mixture of the XANES spectra of reference components, such as metal foil and bulk oxide. This fitting method on the assumption that the XANES signal of atoms collection is the linear sum of the XANES from individual components, which is valid under all conditions except the harsh conditions. In this sense, LCF is a useful approach to XANES analysis, and is generally very easy to perform. Sensitivity can be somewhat limited if done carefully, but can also be quite robust fitting method.

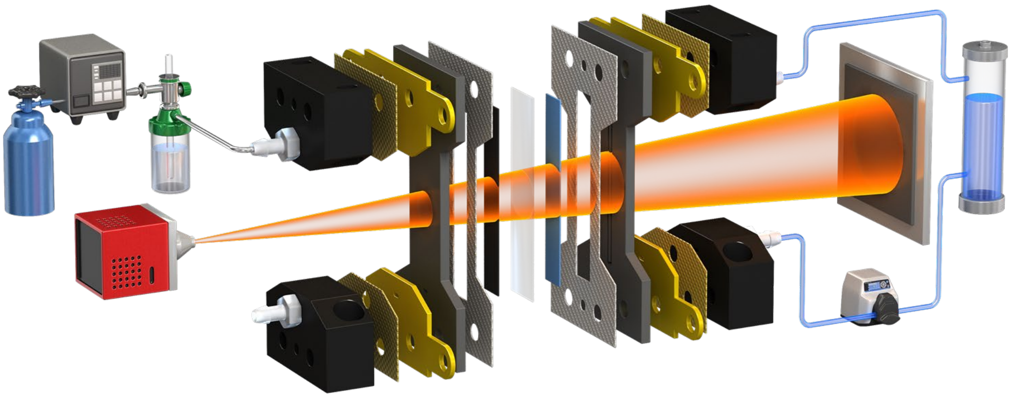

Supplementary Figure 26. System of *in-situ/operando* synchrotron computed tomography (CT) analysis with the homemade electrochemical zero-gap CO_2RR electrolyzer.

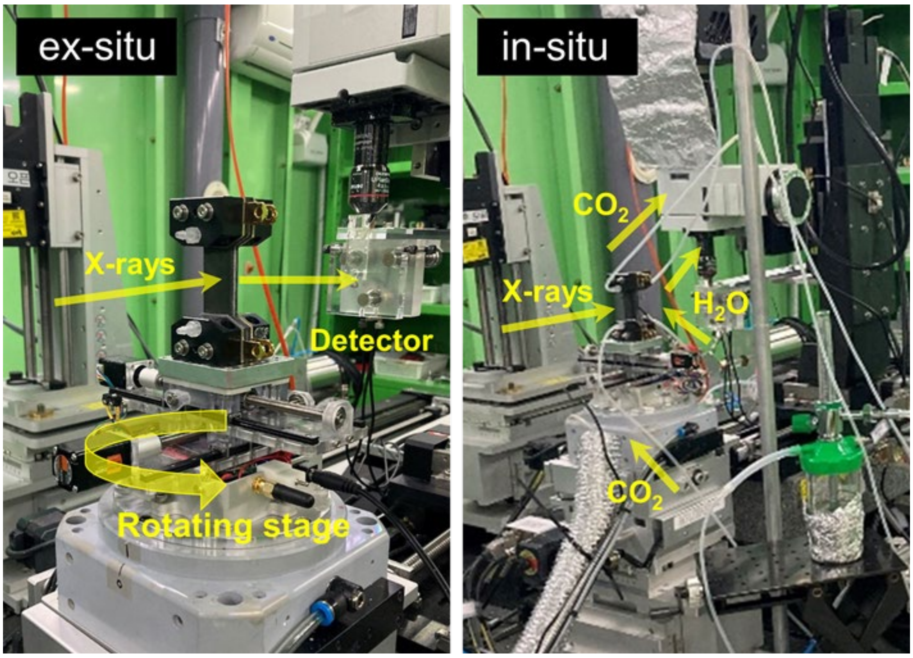

Supplementary Figure 27. Photo of a synchrotron-based X-ray computed tomography (CT) flow cell and system for electrochemical CO_2 reduction reaction (CO_2RR).

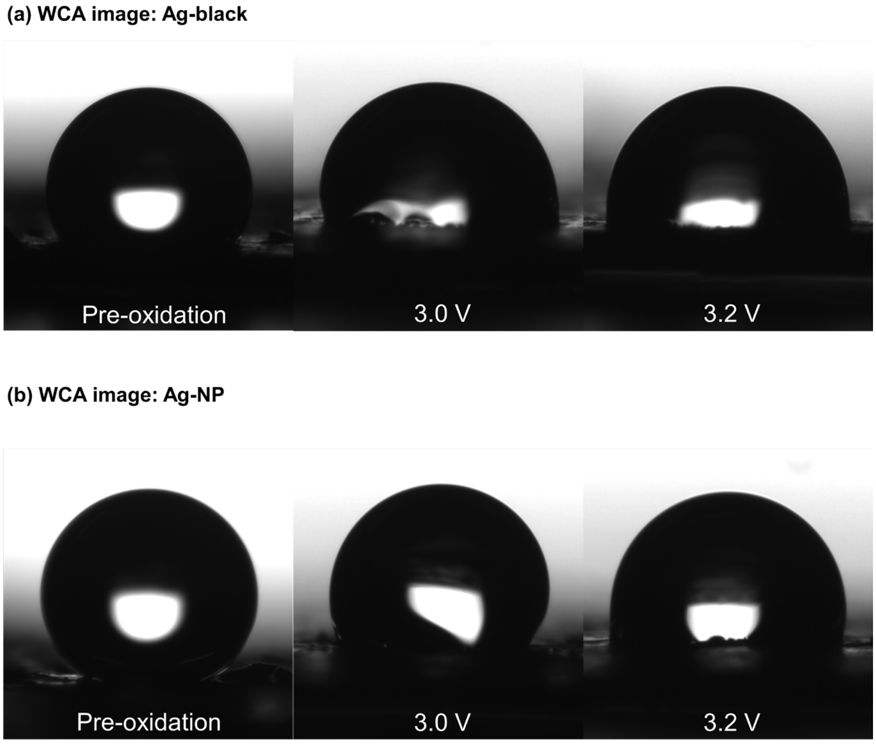

Supplementary Figure 28. Water contact angle (WCA) analysis of (a) Ag black and (b) Ag-NP cathodes after preoxidation and CO_2RR at 3.0 V and 3.2 V, respectively.

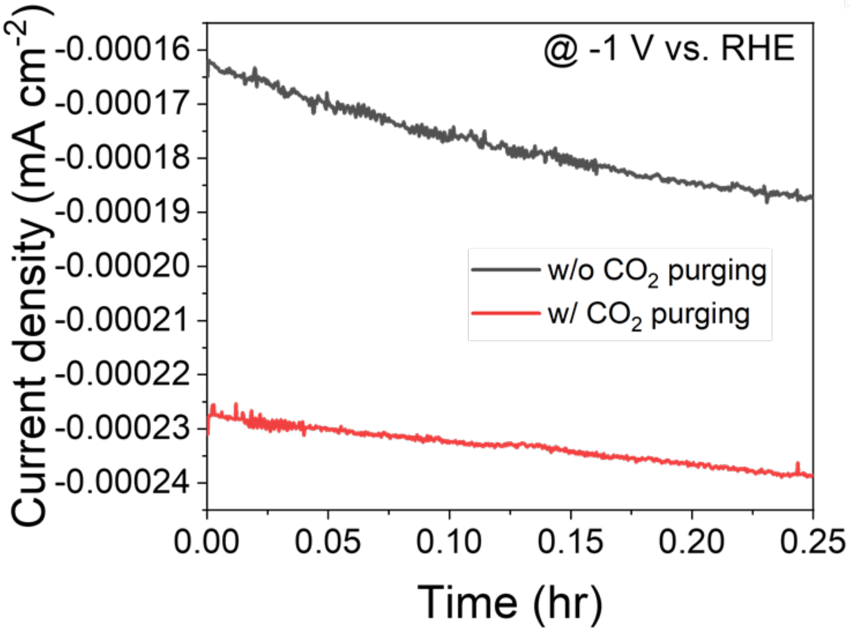

Supplementary Figure 29. Chronoamperometric response recorded in 0.1 M $KHCO_3$ solution at 1.0 V vs Reversible hydrogen electrode (RHE) for bare Au grid.
