# Supplementary material for: Extrinsic hydrophobicity-controlled silver nanoparticles as efficient and stable catalysts for CO2 electrolysis
Source: Nat Commun. 2024 Apr 18;15:3356. doi: 10.1038/s41467-024-47490-3 (PMC11026478; doi:10.1038/s41467-024-47490-3)
Supplement: Supplementary file 3 — Description of Additional Supplementary Files [file 41467_2024_47490_MOESM3_ESM.pdf]

Supplementary Movies 1-6:

Supplementary Movie 1 | Synchrotron Radiation X-ray CT for as-prepared Ag black electrode.

Supplementary Movie 2 | In-situ/*Operando* synchrotron Radiation X-ray CT for Ag black electrode during CO_2RR at 2.8 V.

Supplementary Movie 3 | In-situ/*Operando* synchrotron Radiation X-ray CT for Ag black electrode during CO_2RR at 3.4 V.

Supplementary Movie 4 | In-situ/*Operando* synchrotron Radiation X-ray CT for Ag-NP electrode.

Supplementary Movie 5 | In-situ/*Operando* synchrotron Radiation X-ray CT for Ag-NP electrode during CO_2RR at 2.8 V.

Supplementary Movie 6 | In-situ/*Operando* synchrotron Radiation X-ray CT for Ag-NP electrode during CO_2RR at 3.4 V.
